# Revealing systematic changes in the transcriptome during the transition from exponential growth to stationary phase

**DOI:** 10.1128/msystems.01315-24

**Published:** 2024-12-23

**Authors:** Hyun Gyu Lim, Ye Gao, Kevin Rychel, Cameron Lamoureux, Xuwen A. Lou, Bernhard O. Palsson

**Affiliations:** 1 Department of Biological Sciences and Bioengineering, Inha University, Incheon, South Korea; 2 Department of Bioengineering, University of California, San Diego, California, USA; 3 Joint BioEnergy Institute, Emeryville, California, USA; 4 The Second Hospital of Shandong University, Jinan, Shandong, China; 5 The Novo Nordisk Foundation Center for Biosustainability, Technical University of Denmark, Kgs. Lyngby, Denmark; Danmarks Tekniske Universitet The Novo Nordisk Foundation Center for Biosustainability, Kgs. Lyngby, Lyngby-Taarbæk, Denmark

**Keywords:** stationary phase, systems biology, independent component analysis, nutrient starvation, stress, transcriptome, RNA-sequencing

## Abstract

The composition of bacterial transcriptomes is determined by the transcriptional regulatory network (TRN). The TRN regulates the transition from one physiological state to another. Here, we use independent component analysis to monitor the composition of the transcriptome during the transition from the exponential growth phase to the stationary phase. With *Escherichia coli* K-12 MG1655 as a model strain, we trigger the transition using carbon, nitrogen, and sulfur starvation. We find that (i) the transition to the stationary phase accompanies common transcriptome changes, including increased stringent responses and reduced production of cellular building blocks and energy regardless of the limiting element; (ii) condition-specific changes are strongly associated with transcriptional regulators (*e.g.*, Crp, NtrC, CysB, Cbl) responsible for metabolizing the limiting element; and (iii) the shortage of each limiting element differentially affects the production of amino acids and extracellular polymers. This study demonstrates how the combination of genome-scale datasets and new data analytics reveals the fundamental characteristics of a key transition in the life cycle of bacteria.

**IMPORTANCE:**

Nutrient limitations are critical environmental perturbations in bacterial physiology. Despite its importance, a detailed understanding of how bacterial transcriptomes are adjusted has been limited. By utilizing independent component analysis (ICA) to decompose transcriptome data, this study reveals key regulatory events that enable bacteria to adapt to nutrient limitations. The findings not only highlight common responses, such as the stringent response, but also condition-specific regulatory shifts associated with carbon, nitrogen, and sulfur starvation. The insights gained from this work advance our knowledge of bacterial physiology, gene regulation, and metabolic adaptation.

## INTRODUCTION

Transition to the stationary phase from the exponential growth phase involves significant physiological adaptation for the shift, orchestrated by many regulators for adjustments in gene expression (1). A detailed understanding of this transition is fundamental for comprehending microbial physiology in diverse contexts, including environmental adaptation (*e.g.*, colonization of nutrient-limited niches), infection, and industrial fermentation. Among the many factors influencing this transition, nutrient availability plays a critical role (2); when nutrients are abundant, cells grow exponentially, prioritizing rapid division and biosynthesis, but when nutrients become limited, growth ceases, triggering metabolic reprogramming. These adaptations are critical for microbial persistence in fluctuating ecosystems and host environments, where competition and stressors are prevalent. Therefore, it is critical to manifest the process in regard to how gene expression is coordinately regulated by multiple regulators consisting of the transcriptional regulatory network (TRN).

Although the systematic monitoring of changes in the transcriptome has been limited, independent component analysis (ICA) was recently applied as a knowledge-based approach to the decomposition of bacterial transcriptomes (3, 4). In a recent comparison of different matrix decomposition methods for transcriptomic data, ICA was the best method for detecting known gene groups under the same regulation (5). ICA-identified independently modulated gene groups are called iModulons (3). More specifically, the ICA decomposition of a transcriptome dataset mathematically takes the form of **X = MA** where **X** is the RNA-sequencing data matrix (genes by conditions), **M** is the condition-independent iModulon matrix (genes by iModulons) that contains the signals, and **A** is the matrix of iModulon condition-dependent activities. (*i.e.*, signal strengths, organized as iModulons by conditions). Thus, the columns of **M** give the signals or the lists of genes found in an iModulon. When ICA was applied to *Escherichia coli* transcriptomic data (3), many of the iModulons were shown to represent gene groups regulated by the same regulator(s). Several iModulons lacking associated regulators represent unknown regulatory functions or rearrangements of the genome (such as segmental amplifications, gene deletions, etc). Recently, this effort has been extended to many other microorganisms (*e.g.*, *Bacillus subtilis* [6], *Pseudomonas putida* [4], *Vibrio natriegens* [7]) and generated a database accessible at https://iModulonDB.org (8). iModulonDB is a database of qualified transcriptomes for a strain where a critical number of datasets are available under various conditions to compute the iModularization of its transcriptome.

Recently, the iModulons for *E. coli* K-12 strains have been built through a series of database expansions to include 1,035 RNAseq profiles generated under the same protocol (3, 9). This database, called PRECISE-1K, resulted in 201 iModulons where 117 of them are regulatory iModulons representing target gene groups of 91 regulators (9). Since PRECISE-1K has 533 unique conditions, it can provide a fine-resolution view of the signals found in the *E. coli* transcriptome. Since the ICA approach is scalable, more transcriptomes obtained under controlled conditions can be added to the analyzed dataset to achieve a more fine-grained view of signals from bacterial transcriptomes (3, 9).

In this study, we address how the TRN adjusts the composition of the *E. coli* transcriptome to achieve specific physiological states using the transition from exponential growth to early stationary phase as an example. We collect duplicate time series of transcriptome data during the transition from exponential growth to stationary phase under three nutrient depletion conditions: carbon (C), nitrogen (N), and sulfur (S). Utilizing a novel big data analytic approach that decomposes transcriptomes into co-expressed sets of genes (3) simplifies the interpretation of transcriptomic changes. This approach allows us to elucidate the hierarchy of events that occur during the transition from the exponential growth phase to the early stationary phase.

## RESULTS

### Changes during the transition to the stationary phase vary depending on the limiting element

**Fig 1:**
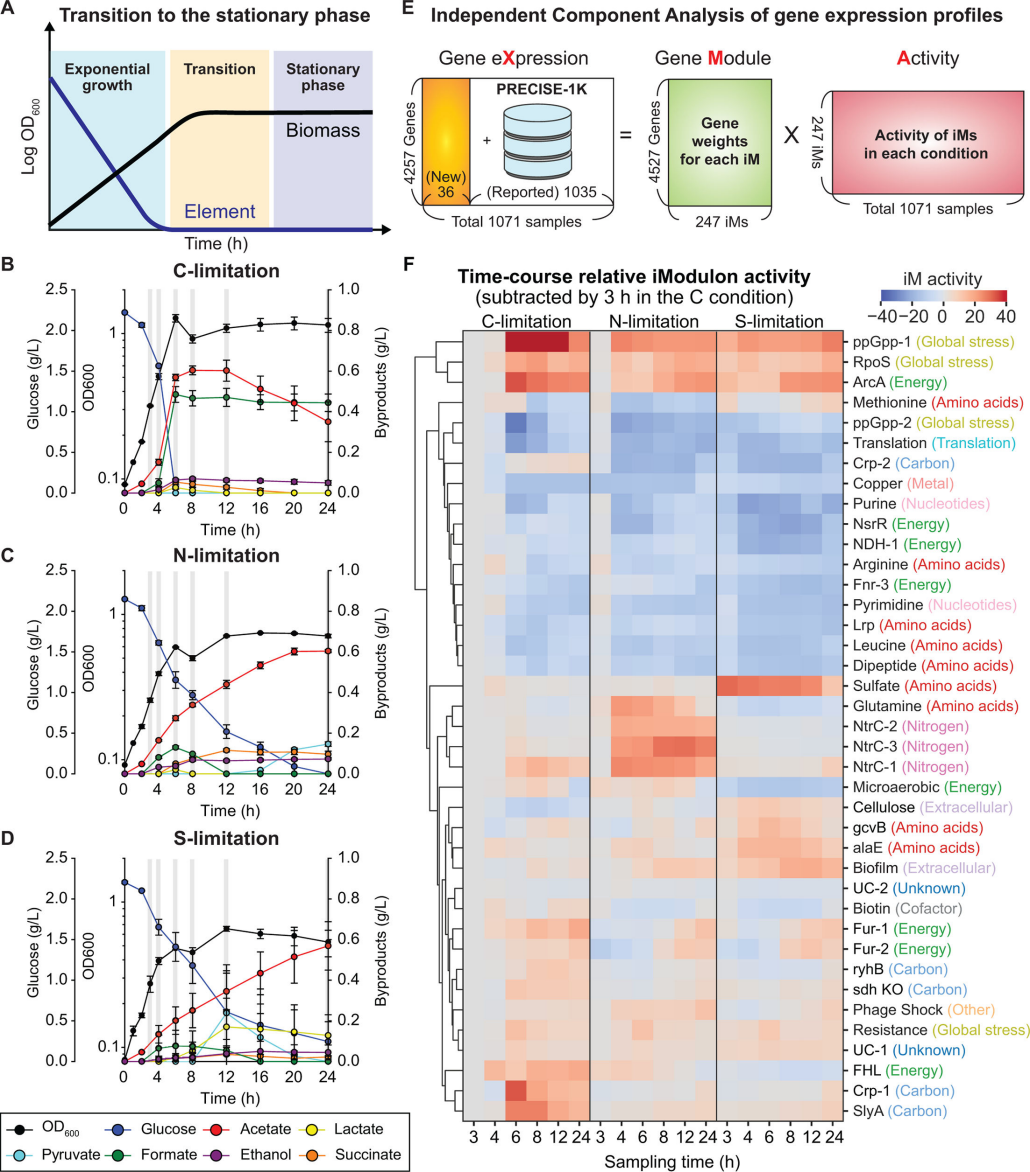
ICA of nutrient limitation-induced transition to the stationary phase. (**A**) A time-course schematic of bacterial growth. (**B-D**) Growth and exo-metabolic data during transition into the stationary phase under (**B**) C, (**C**) N, and (**D**) S limitations. (**E**) Decomposition of a gene expression matrix “X” into a gene weight matrix “M” and an activity matrix “A” for each iModulon (condition-independent groupings of co-regulated genes). ICA was performed for a total of 1,071 transcriptomes, 36 samples generated in this study, and 1,035 samples from PRECISE-1K (9). (**F**) Time-course relative activity of 39 iModulons with explained variances greater than 0.5% as a threshold. iModulons were hierarchically clustered with their activities. All activities were normalized by the activity at 3 h in the C-limiting condition. Each color indicates the category of iModulons.

We carried out a batch culture of *E. coli* K-12 MG1655 in a minimal M9 medium under three different conditions where C, N, and S becomes the limiting element (see **Methods**). The cell growth, glucose consumption, and formation of fermentation byproducts (*i.e.*, pyruvate, lactate, acetate, ethanol, succinate, and formate) were monitored for 24 h (Fig. 1A through D). Cells grew exponentially during the first 6 h under all conditions, with similar maximum specific growth rates: 0.44 h^−1^, 0.35 h^−1^, and 0.39 h^−1^ in the C-, N-, and S-limiting conditions, respectively. After 8 h, no significant increase in OD_600_ was observed across all conditions, indicating that the cells had entered the stationary phase. Although maximum OD_600_ values varied between 0.7 and 1.4, similar amounts (up to 0.6 g/L) of acetate were produced from glucose consumption (Fig. S1). In the C-limiting condition, acetate reassimilation was observed, in addition to a relatively higher accumulation of formate (0.47 g/L). In the N-limiting condition, succinate accumulation was higher (0.1 g/L), whereas lactate accumulation (0.13 g/L) was elevated in the S-limiting condition. These observations imply that each element-limiting condition exerts both common and specific effects on cellular physiology and gene expression.

We obtained 36 RNA-seq gene expression profiles across the three conditions at six time points with two biological replicates to understand transcriptional regulatory events during the transition. The replicate profiles showed high Pearson R values, with a median of 0.97, clearly distinct from the values obtained by randomly choosing non-replicate profiles (Fig. S2A). With the C-limited condition at 3 h as a reference, 3,747 genes (86.7% of the 4,322 total genes) displayed differential gene expression (fold change >2 with p_adj_ < 0.05) in at least one pairwise comparison, indicating that the transition into the stationary phase led to a global reallocation of the transcriptome.

We utilized principal component analysis (PCA) to assess the information content of the gene expression profiles in both sample and gene spaces. Effectively, nine principle components (PCs) explain 90% of the total variance in this dataset (Fig. S2B). The two major PCs in the experimental condition space show distinctive trajectories of the gene expression changes, depending on limiting elements rather than sample collection times (Fig. S2C). The most significant transcriptome changes occurred during the late exponential phase (3–6 h). Interestingly, the transcriptomes in the C-starvation condition at 12 h and beyond became closer to the transcriptomes at the early time point (4 h) of the N-starvation condition. This was likely due to the re-assimilation of secreted acetate, indicating that N became an additional limiting nutrient in this condition. In contrast, changes in the transcriptomes under the N- and S-starvation conditions remained similar even after 12 h, likely because no alternative nutrients were available in the media.

A PCA in the gene space was also performed and showed that the first four PCs explain more than 90% of the total variance (Fig. S2D). Most genes were clustered at the center in the plane of the two major PCs, which explains 79.7% and 10.5% respectively, and approximately 20% of genes were divergently located. A few gene clusters showed a high correlation in their expression. These genes are expressed from the same operons (*e.g.*, *carA* and *carB* encoding the carbamoyl-phosphate synthetase small and large subunit, respectively) or involved in the same metabolic pathways (*e.g.*, *uraA* encoding uracil transporter UraA). However, further interpretation was difficult given that the number of distinctive clusters and their sizes were small. Furthermore, understanding regulatory events during the transition from the analysis was limited given that PCs lack their associations with biological events. Although the PCA in the sample space showed that the starvation of each element resulted in distinctive transcriptional responses, the PCA in the gene space required an alternative analysis for a comprehensive understanding of regulatory events, resulting in transcriptome changes during the transition.

### ICA identifies key common transcriptome changes governed by global transcriptional regulators

We performed ICA for the 1,071 total gene expression profiles (1,035 plus 36 from PRECISE-1K and this study; see Methods for the ICA calculation, Fig. 1E). Although the most recent *E. coli* iModulon structure was built based on analyzing more than a thousand samples, a new iModulon structure was re-calculated to obtain a better resolution for the newly generated samples. We obtained 247 iModulons that explain 82.5% and 78.2% of the total variance within the 1,071 gene expression profiles and the 36 stationary-phase gene expression profiles, respectively (Fig. S3A). With the new calculation, the iModulon size was increased by 46 from 201 in the previous study (9), and the explained total variance was alsAQere robustly observed (Pearson R of 191 iModulons > 0.7, Fig. S3B). For less-correlated iModulons, their potential regulators were enriched from the TRN information and named by an enriched regulator if present (see Materials and Methods).

We identified 3Dd9 iModulons, associated with 28 regulators, that explain at least 0.5% of the variances in the dataset (Fig. 1F; Fig. S4). Many of the regulators were global regulators, such as RpoS, ppGpp, Crp, Fur, and ArcA, known to be important for the stationary phase response (10). This observation implies that using ICA for the dataset successfully identified major changes, consistent with previous studies. Additionally, some iModulons (ppGpp-1, alaE, NsrR, ryhB, and SlyA) were not conserved in the last iModulon structure, suggesting potential discoveries. Large activity changes of the 39 iModulons represent the signature changes in the transcriptome during the transition. Consequently, we focused on analyzing their time-course activity changes and exploring correlations between them.

#### ppGpp orchestrates a reallocation of a global transcriptome during the transition

**Fig 2:**
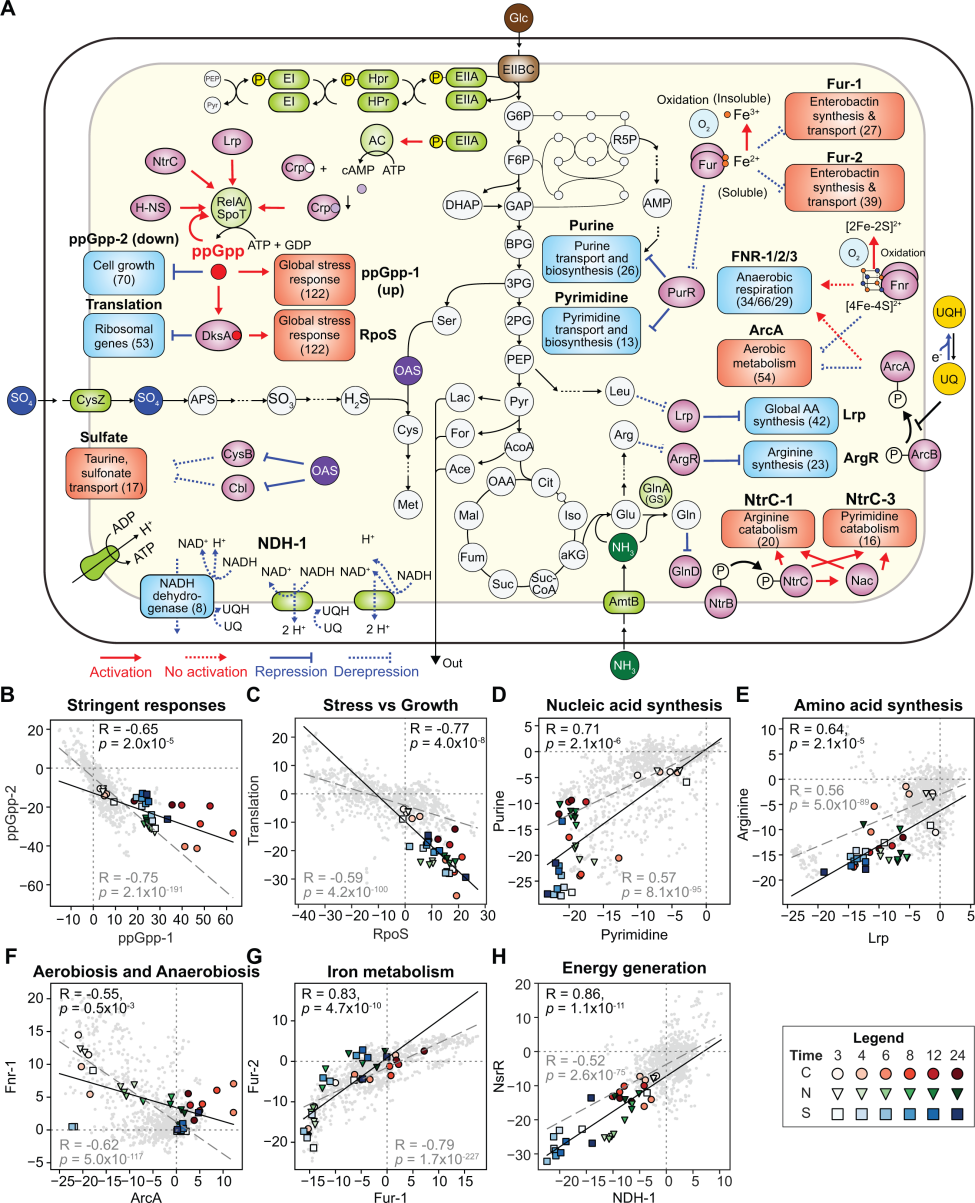
Activities of iModulons obtained from ICA for the 36 stationary-phase RNAseq samples. (**A**) A map that outlines the functions of iModulons with the strongest changes in activities during the transition to the stationary phase. (**B-H**) Compared iModulon activities of the 14 representative iModulons: (**B**) ppGpp-1 vs ppGpp-2, (**C**) RpoS vs Translation, (**D**) Pyrimidine vs Purine, (**E**) Lrp vs Arginine, (**F**) ArcA vs Fnr-1, (**G**) Fur-1 vs Fur-2, and (**H**) NDH-1 vs NsrR. Black lines and grey dotted lines indicate correlations among the 36 samples and the entire samples, respectively. Pearson correlations (R) and corresponding *P*-values were given. Symbols: red circles, C-limiting; green inverted triangles, N-limiting; blue squares, S-limiting.

We found that the activities of two ppGpp-associated iModulons (ppGpp-1 and ppGpp-2) displayed significant changes across conditions but with a negative correlation (Pearson R = −0.65 at *P* = 2.0 ✕ 10^−5^, Fig. 2A and B). ppGpp (guanosine tetraphosphate) is a key alarming molecule, synthesized by the RelA/SpoT homolog family (RSH). It is known to regulate translation and transcriptional processes during stringent responses, such as nutrition starvation by directly interacting with RNA polymerases (10, 11). The ppGpp-1 iModulon contains 46 genes with greatly increased expression at starvation. Its member genes include genes encoding stress-induced proteins (*e.g.*, YchH, UspB) and toxin–antitoxin systems (*e.g.*, MqsR-MqsA, RelB-RelA [12, 13]). The ppGpp-2 iModulon contains 82 genes, and many genes are related to translation, ribosomal structure and biogenesis, coenzyme and nucleotide transport, and metabolism (Fig. S5). These two iModulons with opposite activities capitulate the role of ppGpp as an activator and a repressor.

We also observed a strong correlation between the RpoS and translation iModulon (Pearson R = −0.77 at *P* = 4.01 ✕ 10^−8^, Fig. 2C), which are also regulated by ppGpp and DksA but in a different mode (Fig. 2A). The RpoS iModulon includes 116 genes and its many member genes are responsible for cellular responses to diverse stressors (*e.g.*, pH, osmotic stress) and regulated by RpoS (3). The translation iModulon contains 51 genes, mostly encoding ribosomal subunit genes, responsible for translating mRNA to proteins. This global negative correlation is conserved in many bacteria and is often called the “fear-greed trade-off” (3, 4, 14, 15). This iModulon analysis confirmed that ppGpp and related regulators governed global transcriptome reallocation for shifting growth phenotypes to maintenance to maximize cellular persistency regardless of the limiting elements.

#### Nutrient starvation results in cessation of nucleotide and amino acid metabolism

We identified huge changes in the activities of iModulons (*e.g.*, Pyrimidine, Purine, Lrp, Arginine) related to nucleotide and amino acid metabolism (Fig. 2D and E). The pyrimidine and purine iModulons contain 13 (*e.g.*, *carAB*, *pyrIB*) and 26 (*e.g.*, *purKE*, *purMN*, *xanP*) genes, respectively. PurR mainly regulates the member genes of both iModulons. The Lrp and Arginine iModulons contain 22 (*e.g.*, *livFGMHK*) and 23 genes (*e.g.*, *argA*, *argG*, *argD*, *argECBH*, *argI*), respectively. These iModulons are mainly regulated by Lrp and ArgR and are associated with nucleotide and amino acid metabolism. The reduced activities indicate a slowdown in the production of the key cellular building blocks. Interestingly, the activities of some iModulons differentially changed depending on limiting nutrients. For example, the arginine iModulon activity increased after 8 h in the C- and N-limiting conditions, while it remained low in the S-limiting condition. These results suggest multiple layers in the regulation or potential fluctuation of nutrient pools from scavenging premade building blocks.

#### Activity changes of redox state-related iModulons indicate oxidation of the intracellular environment during the transition

During the transition to the stationary phase, iModulons related to intracellular redox status (*e.g.*, ArcA and Fnr) displayed similar trends, regardless of the conditions (Fig. 2F and G). ArcA forms a two-component system together with membrane-associated kinase ArcB and responds to the availability of reducing cofactors (*e.g.*, ubiquinol, Fig. 2A) (16). When ubiquinol is abundant, ArcB transfers a phosphate group to ArcA. The resulting ArcA-P suppresses the expression of aerobic metabolism genes and activates anaerobic respiration genes (17). Conversely, in an oxidized environment, ubiquinol is oxidized to ubiquinone, and the activity of ArcB is inhibited, resulting in the upregulation of aerobic metabolism genes. On the other hand, Fnr (fumarate and nitrate reduction regulator) also plays a significant role, depending on the redox state of iron–sulfur clusters. Iron–sulfur clusters can exist in multiple forms (*e.g.*, [4Fe-4S]^2+^, [2Fe-2S]^2+^). In a reduced environment, Fnr binds to [4Fe-4S]^2+^ and the resulting form activates anaerobic respiration-related genes (18). When [4Fe-4S]^2+^ is oxidized to [2Fe-2S]^2+^, Fnr loses the activation function. The activity of AcrA and Fnr iModulons displayed a negative correlation (Fig. 2F), where the activity of the ArcA iModulon generally increased, and the activity of the Fnr-1 iModulons decreased during the transition. These changes in their activities show that the intracellular environment was transiently oxidized, likely owing to reduced carbon assimilation and subsequent lower reducing cofactor generation.

The oxidation was also supported by increased activities of two Fur-related iModulons (Fur-1 and Fur-2, Fig. 2G). Fur is a TF, which controls iron metabolism depending on iron availability (19). Iron ions are relatively plentiful in a reduced environment since iron presents as water-soluble ferrous ions (Fe^2+^). In this condition, Fur bound by Fe^2+^ suppresses the expression of enterobactin synthesis genes responsible for water-insoluble ferrite ions (Fe^3+^) uptake. Therefore, the increased iModulon activities indicate iron starvation because of the gradual oxidation of the intracellular environment. Collectively, the activity changes of the redox-related iModulons consistently indicated that the intracellular environments are transiently oxidized, regardless of experimental conditions.

#### Oxidative phosphorylation is reduced in the stationary phase

Two iModulons, NDH-1 and NsrR, displayed largely decreased activities during the transition to the stationary phase (Fig. 2H). The NDH-1 iModulon contains eight key genes consisting of the electron transport chain (*nuoGHIJKLMN*). From its low activity, it was inferred that energy generation is less active in the stationary phase. On the other hand, the NsrR iModulon is a nitrite-sensitive repressor, and its iModulon contains eight member genes: *rsxDGE* encoding SoxR reducing protein, *wecC-rffGHC* encoding cell-surface antigen synthesis genes, and *recG* encoding ATP-dependent DNA helicase. Low expression of the SoxR-related genes suggested that oxidative stresses were less induced in starvation conditions, likely owing to the reduced metabolism. Interestingly, the activities of these two iModulons were particularly low in the N- and S-starvation conditions. Given fermentation products, such as lactate and succinate, were relatively more accumulated in these conditions (Fig. 1B through D), it was also inferred that the activity of the electron transport chain was reduced.

### ICA shows sophisticated regulation of multiple TFs for gene expression for different element starvation

We investigated iModulon activity changes specific to each element-limiting condition (Fig. 1F; Fig. S4). Many of the condition-specific iModulons with high activity changes are related to Crp, NtrC (also known as GlnG), CysB, and Cbl which are global regulators for the metabolism of C (Crp) (20), N (NtrC) (21–23), and S (CysB and Cbl) (24). Generally, the expression of these regulator genes was condition, specifically different in the corresponding nutrients (Fig. S6). However, Cbl expression was also affected in the N-limiting condition in addition to the S-limiting condition likely because it is regulated by the level of adenosine 5′-phosphosulfate requiring nitrogen.

#### Glucose starvation affects the activity of Crp-related iModulons responsible for alternative carbon metabolism

**Fig 3:**
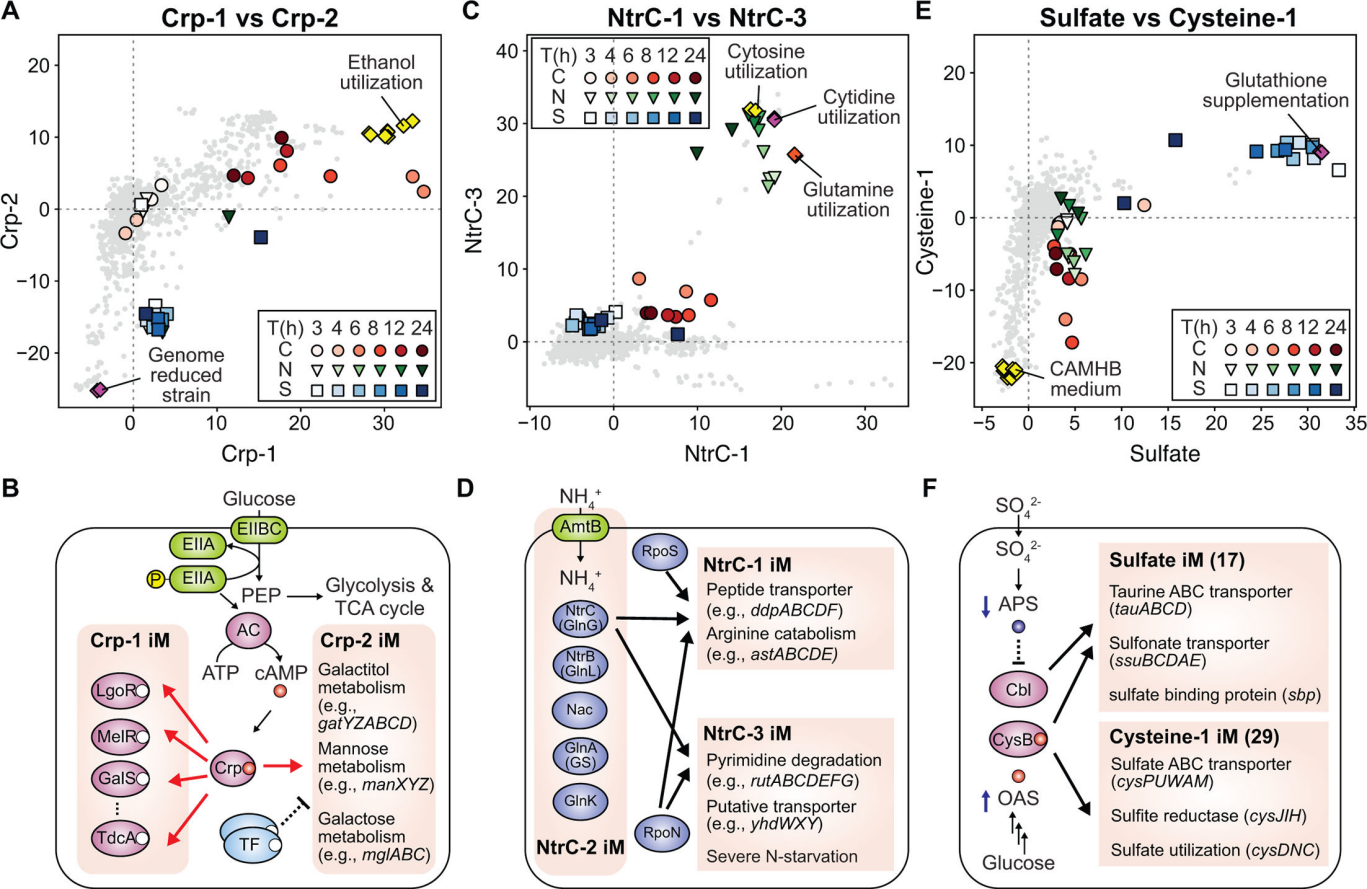
Distinctive global transcriptome changes depending on starving nutrients. (**A-F**) Activities, functions, and related regulators for iModulons related to the C, N, and S metabolism. (**A**) Activity changes of the Crp-1 and Crp-2 iModulons. iModulon activities of PRECISE-1K samples under ethanol utilization (5 g/L) as a sole carbon source, and genome-reduced strains were additionally indicated. (**B**) Regulation of Crp for the two iModulons. (**C**) Activity changes of the NtrC-1 and NtrC-3 iModulons. iModulon activities of PRECISE-1K samples under the utilization of either cytosine, cytidine, and glutamine as a N source were additionally indicated. (**D**) Regulation of NtrC for the two iModulons. (**E**) Activity changes of the sulfate and cysteine-1 iModulons. iModulon activities of PRECISE-1K samples in the CAMHB medium or with the supplementation of glutathione were additionally indicated. (**F**) Regulation of Cbl and CysB for the two iModulons. Symbols: red circles, C-limiting; green inverted triangles, N-limiting; blue squares, S-limiting.

We found that the activities of the Crp-1 and Crp-2 iModulons greatly changed in the three conditions but did so differently, depending on the limiting nutrients (Fig. 1F and 3A). Crp controls carbon metabolism by sensing cyclic-AMP (cAMP), whose synthesis is activated by the degree of phosphorylation of the EIIA component of the phosphotransferase system (PTS, Fig. 3B). The Crp-1 iModulon contains transcription factor genes, such as *lgoR*, *galS, melR*, and *tdcA* (Fig. S7A). These genes encode activators for catabolism genes by sensing galactonate, galactose, mellobiose, and threonine, respectively. The activity of the Crp-1 iModulon increased as glucose was depleted, which implies that TF genes were expressed to seek alternative carbon sources rather than being constitutively expressed. Such high activities were observed in samples collected when ethanol was utilized as a carbon source, which is likely a similar glucose-starvation condition. Interestingly, its activity decreased once acetate was re-assimilated. This observation was likely due to a higher cAMP level since acetate is not assimilated *via* the PTS system.

In contrast, the activity of the Crp-2 iModulon was maintained at similar levels in the C-limiting condition only where its activity decreased in the N- and S-starvation conditions (Fig. 3A). This iModulon contains 20 genes encoding transporters or metabolic enzymes for alternative sugars (*e.g.*, *gatYZABCD* for galactitol, *manXYZ* for mannose, *mglABC* for galactose, Fig. S7B). This iModulon also displayed increased activity in the C-limitation condition, which is consistent with its upregulation by cAMP-Crp (25). However, it commonly decreased in the N- and S-limiting conditions to a very low level; similar to a level in a *gatABCDE*-deleted *E. coli* strain (DGF-298, a reduced genome strain) (26). The specific high activities of the two iModulons in the C-limiting condition show precise regulation associated with CRP for the carbon metabolism.

#### NtrC-related iModulons are sophisticatedly regulated for a different activation depending on limiting nutrients and time points

In the N-limiting conditions, three NtrC-related iModulons (NtrC-1, NtrC-2, and NtrC-3) showed large activity changes, indicating that their member genes are responsible for N-related regulation (Fig. 1F and 3C) (27). NtrC (GlnG) is known as a primary regulator for nitrogen metabolism together with Nac, which are known to regulate 50 and 519 genes, respectively (Supplementary Data). The NtrC-1 iModulon contains *astABCDE* genes responsible for dipeptide transport (*ddpABCDF*) and NH_3_-generating arginine catabolism (Fig. 3D; Fig. S7C). The NtrC-2 iModulon includes nitrogen-related regulator genes (*e.g.*, *glnK*, *glnL*, *ntrC*, *nac*) and transporter genes (*e.g.*, *amtB*, Fig. S7D), while the NtrC-3 iModulon includes *rutABCD* for pyrimidine degradation and *yhdWXZ* with putative transporter for an unknown substrate (Fig. S7E). Commonly, the activities of these three iModulons increased and maintained until the end of the cultivation at high levels. This observation was different from the returned activity of the Crp-1 iModulon after initiating acetate assimilation in the C-limiting condition; it was likely because an alternative nitrogen source was unavailable in the medium.

The generation of the three different iModulons for NtrC-regulon genes implies that independent signals are present in the dataset (Fig. S8A through C). Indeed, it was noticed that they display different activities depending on conditions and time points. Specifically, while NtrC-1 activity reached the maximum level at 4 h, the activity of NtrC-3 was the greatest at 12 h. Moreover, the NtrC-1 iModulon activity was also increased in the C- and S-limiting conditions, whereas the activities of the other iModulons did not similarly increase. We believe that such different behaviors are attributed to the separation of the NtrC regulons into multiple iModulons and potential regulation by other co-regulators. For example, RpoS was enriched as a co-activator for the NtrC-1 iModulon, containing genes involved in amino acid transport and catabolism. Its activation by RpoS is perhaps due to potential beneficial effects in a stress condition, not limited to N-starvation, in relieving stress. On the other hand, the activity of the NtrC-3 iModulon containing many genes encoding enzymes responsible for degradation of nucleic acid building blocks (*e.g.*, *rutABCDEFG* for pyrimidine degradation, Fig. S7E) were highly specific to severe N starvation. Given it is potentially more detrimental to cells when excessively expressed compared to degradation of proteogenic amino acids, these different and sophisticated regulations by multiple TFs might imply that cells utilize dose-dependent strategies to obtain nitrogen sources for survival. Indeed, both activities of NtrC-1 and NtrC-3 were different depending on nitrogen sources in samples from a previous study (27) included in PRECISE-1K (Fig. S8). The activities of the NtrC-3 iModulon were higher when either cytosine or cytidine was utilized as a nitrogen source compared with glutamine, whereas the NtrC-1 iModulon activity was higher in the glutamine condition (Fig. 3C).

#### Cbl and CysB are two major regulators for transcriptome changes in S-limiting conditions

In the S-limiting condition, we observed an immediate increase in the activity of the sulfate iModulon, which is co-regulated by Cbl and CysB (Fig. 1F and 3E), both of which are major regulators for sulfur metabolism. Cbl upregulates the gene expression when sulfate is not readily available, which is detected by a level of adenosine 5-phosphosulfate (APS) containing a sulfur atom (Fig. 3F) (28). On the other hand, CysB represses its target genes in the absence of O-acetyl-serine (OAS), an intermediate in the cysteine biosynthesis pathway that utilizes sulfur as a substrate, and activates them when OAS is accumulated due to a shortage of sulfur (29) (Fig. 2A). The sulfate iModulon contains a total of 17 genes, including *tauABCD* and *ssuEADCB* for taurine and sulfonate transporter genes (Fig. S7F). Its highest activation was observed at 3 h (Fig. 1F; Fig. S8D). This high activity was exceptional in the PRECISE-1K dataset; such activity was observed in only a few conditions (*e.g.*, glutathione supplementation, Fig. 3E). Interestingly, its activity gradually decreased over time. Additionally, although an activity increase was smaller, another CysB-related iModulon (Cysteine-1) containing 29 genes (Fig. S7G and S8E) also displayed changed activity in this condition. This iModulon contains *cysPUWAM* encoding sulfate/thiosulfate transporter subunits and *cysDNC* encoding sulfate adenylyltransferase subunits. Its activity was low when cells were grown in a rich medium (*e.g.*, CAMHB medium), where S-containing amino acids are readily available. Interestingly, we noticed that the activity of the cysteine-1 iModulon was affected by C and N starvation. Its activity decreased during the transition phase and increased to the unaltered level. This observation was likely because of a potentially reduced availability of OAS, whose synthesis requires C and N elements. Taken as a whole, ICA successfully captured how the transcriptome is re-organized with global element-metabolism regulators and their expression changes.

### ICA captures unexpected transition-induced changes depending on limiting elements

Since ICA does not rely on pre-existing knowledge, it is useful for discovering new findings from unexpected gene expression changes. As examples, two less-studied features of the transition to the stationary phase are introduced, from an investigation of unexpected iModulon changes and their correlations.

#### Expression of amino acid synthesis genes are affected differently depending on limiting elements

**Fig 4:**
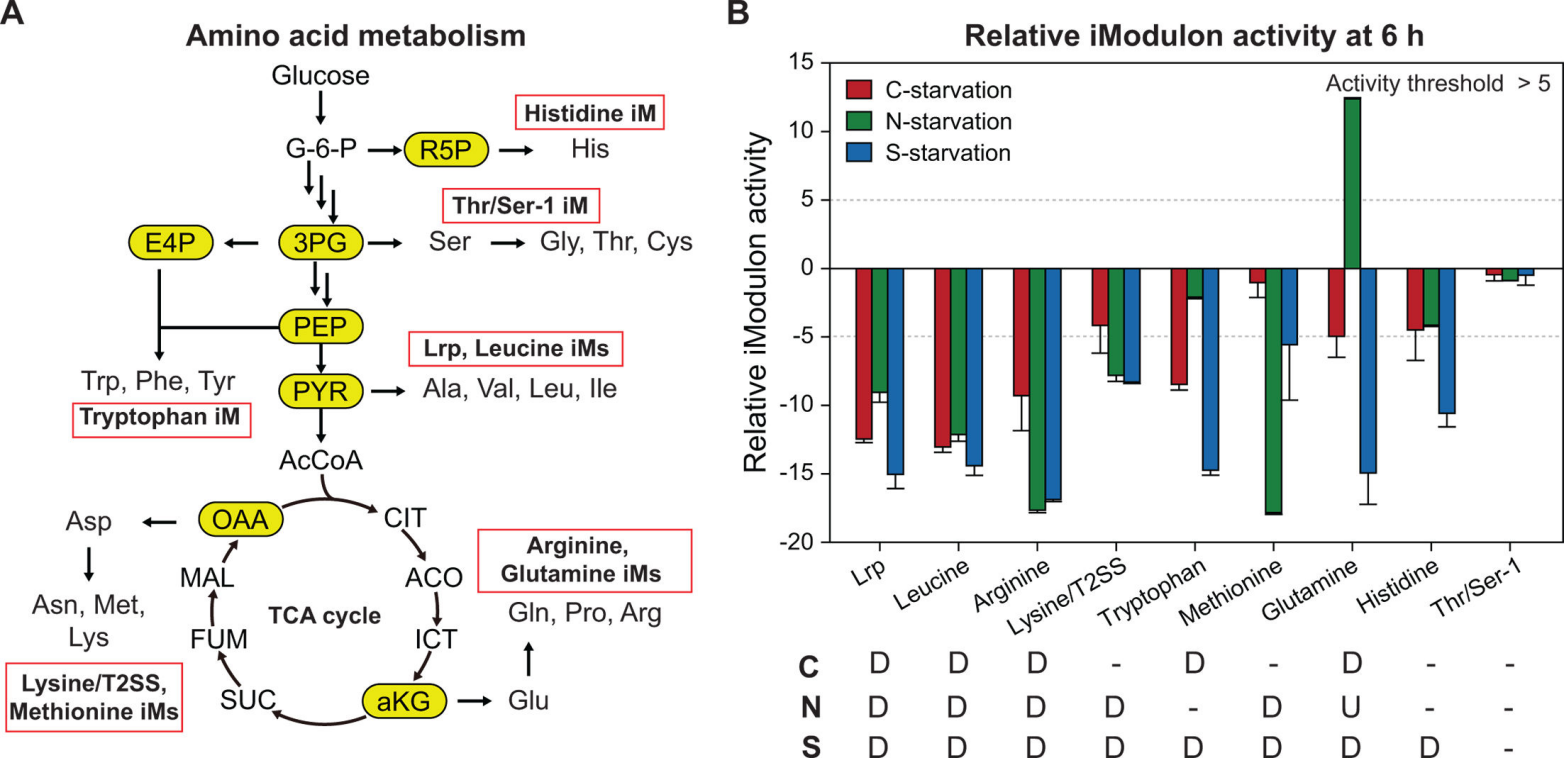
Different activity changes of amino acid metabolism-related iModulons. (**A**) Simplified metabolic pathway map for amino acid metabolism and related iModulons. (**B**) Relative iModulon activity at 6 h compared with their activities in the C-limiting condition at 3 h. “D”, “U”, and “-” indicates “downregulation,” “upregulation,” and “no change,” respectively, with an activity threshold of 5.

Although amino acid synthesis-related iModulons displayed generally reduced activities, their changes varied depending on limiting elements (Fig. 4A and B). Lrp, Leucine, Arginine, and Lysine/T2SS displayed similar activity decreases in the three conditions. However, the other iModulons showed unchanged or oppositely changed activities in at least one condition. For example, the activity of the Tryptophan iModulon showed negligible changes in the N-starvation condition, whereas it was greatly altered in the other conditions. Conversely, the methionine iModulon displayed the highest activity decrease in the N-starvation condition compared with the other conditions. The glutamine iModulon activity increased in the same conditions. These observations indicate that the synthesis of all amino acids has not ceased even in the transition to the stationary phase, and there are less-characterized additional regulation in amino acid synthesis depending on limiting elements. For example, the expression of the Tryptophan iModulon genes can be maintained by alternative activation under N starvation by a related global regulator(s) (27) or small regulatory RNA (30).

#### Sulfur and carbon limitation oppositely affect extracellular polymer synthesis

**Fig 5:**
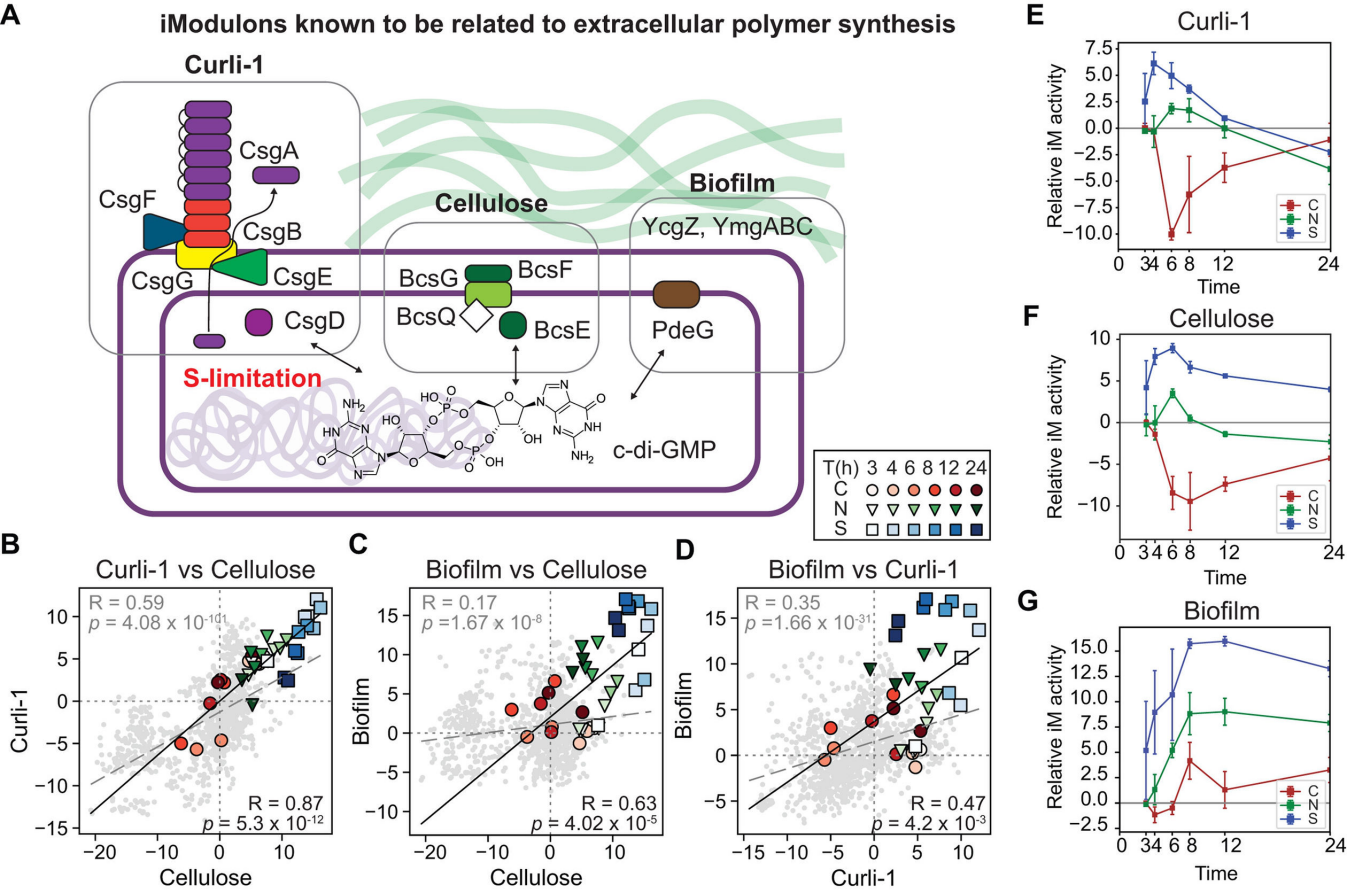
Activities of iModulons known to be related to extracellular polymer synthesis. (A) A schematic diagram of gene functions in the three iModulons (Curli-1, cellulose, and biofilm) related to extracellular polymer synthesis. (B-D) Activity comparisons of between two of the three iModulons: (B) Curli-1 vs cellulose; (C) biofilm vs cellulose; (D) biofilm vs Curli-1. Black lines and grey dotted lines indicate correlations among the 36 samples and the entire samples, respectively. Pearson correlations (R) and corresponding *P*-values were given. (E-G) Time-course relative activities of (E) the Curli-1, (F) cellulose, and (G) biofilm iModulons compared with their activities in the C-limiting condition at 3 h. iM, imodulons.

We found activity changes of three iModulons, Curli-1, cellulose, and biofilm, related to the production of extracellular polymers (Fig. 1Fand Fig. 5A). Although the *E. coli* K-12 MG1655 laboratory strain is not known to produce biofilm (31), several related genes remain. Curli are bacterial amyloids and facilitate adhesion to surfaces, biofilm formation, and cell aggregation (32); the Curli-1 iModulon contains six genes forming two operons, *csgAB* and csg*DEFG*, which encode Curli assembly components or its regulator (33–35). The cellulose iModulon contains 81 genes, and some of its high gene-weight genes include bacterial cellulose synthesis-related genes (*e.g.*, *bcsEFG*, *bcsQ*) (36, 37). Finally, the biofilm iModulon contains five genes (*ycgZ-ymgABC-pdeG*). Although the roles of these genes were not fully elucidated, it was reported that *ymgB*, also known as *ariR*, encodes a putative regulator for acid resistance and biofilm formation genes (38, 39). The activities of these three iModulons display concurrent positive correlations in the 36 gene expression profiles (R = 0.87 and *P* = 5.3 ✕ 10^−12^ between Curli-1 and cellulose; R = 0.63 and *P* = 4.0 ✕ 10^−5^ between cellulose and biofilm; R = 0.47, *P* = 4.2 ✕ 10^−3^ between Curli-1 and biofilm, Fig. 5).

Interestingly, each limiting element affected the activities of the three iModulons differently (Fig. 5). Both iModulons showed increased activities in the S-limiting condition but decreased activities in the C-limiting condition. The N limitation did not greatly alter their activities. Across the 1,036 PRECISE samples, the activity of the Curli-1 iModulon was highest in the S-limiting condition (Fig. 5), implying that S limitation is the critical condition inducing extracellular polysaccharide production. The specific activation of these iModulons suggests that S limitation is a vital condition promoting the synthesis of extracellular polymers. Indeed, a recent study indicated that bacterial colonization is a strategy to access a sulfur reservoir of a host (40). For this response, cyclic-di-GMP(c-di-GMP) can function as a signaling molecule, and the three iModulons commonly contain at least one gene (*e.g.*, *csgD*, *bcsE*, *pdeG*) closely related to c-di-GMP. Indeed, the critical roles of c-di-GMP in biofilm formation (41, 42) have been reported.

## DISCUSSION

In this study, we monitored how the transcriptome composition changes during the transition from exponential growth to the early stationary phase. In particular, to obtain a high resolution, we added the 36 newly generated transcriptome samples to 1,035 existing high-quality *E. coli* transcriptome dataset (9) and performed an ICA for the consolidated dataset. By identifying iModulons displaying high activity changes, we captured major changes in transcriptomes and associated 28 regulators. Our data and analytics allowed a comparison of expression changes between three different limiting elements.

There also have been several attempts to systematically understand the transition to the stationary phase for several microorganisms, such as *E. coli* (43), *Bacillus subtilis* (44), *Rhodobacter sphaeroides* (45, 46), and *Bifidobacterium Longum* (47). These studies mainly analyzed the expression of individual genes. For example, one study reported changed expression of stress (*e.g*., *psp* genes) and growth-related genes (*e.g.*, *araFGH*, *fruBKA*) during the transition (43). Alternatively, genes were often clustered based on gene ontology or KEGG pathway for contextualization of transcriptome changes (45). Although these approaches are useful, they could lack explanation associated with regulators and are limited in terms of scale, especially when multiple experimental conditions need to be compared. Conversely, since iModulons are gene groups likely under the same regulation for the same functions, an iModulon activity comparison can simultaneously provide a contextual and regulatory illustration of transcriptome changes.

From the ICA of the newly generated dataset, multiple characteristics of the transition were elucidated. Although “stationary” implies a status of no change, this study shows that the transcriptome is dynamically adjusted to strive against diverse stresses from nutrient depletion. Most importantly, lack of any elements appeared to result in the common stringent responses orchestrated by the level of ppGpp (Fig. 2). These responses include growth cease for resource-saving and expression of machinery responding to intracellular environmental changes. Particularly, transient intracellular oxidation was inferred from the transcriptomes during the transition. Such changes in transcriptomes are aligned with previous reports about nutrient starvation-induced oxidative stresses (1, 48), enhanced cross-resistance to stresses (48, 49), and oxidation of proteomes during the stationary phase (50).

Meanwhile, key regulators for limiting nutrient metabolism activate the expression of genes responsible for uptake and metabolic by-product degradation to overcome low nutrient levels. In case of the glucose depletion, increased gene expression immediately returned to the original level after an alternative carbon source (*i.e.*, acetate) was found and utilized. On the other hand, the expression of N and S metabolism genes remained at high levels because no other alternate substrates were available from media as element supplements.

The iModulon analysis was also effective to discover unexpected changes during the transition. We observed differential patterns of activity changes of the Tryptophan, Methionine, and Glutamine iModulons (Fig. 4). The Tryptophan and Methionine iModulon activities did not change and remained at similar levels in the N- and C-starvation conditions, respectively. The Glutamine iModulon activity was upregulated by the N starvation in contrast to its decreases in the other two conditions. These observations perhaps imply that the maintained or higher expression is crucial for sustaining viability of the cells. Interestingly, decreased activities of most of the amino acid related iModulons were observed during S-starvation, potentially suggesting that S-starvation is relatively more challenging than C or N starvation. Indeed, higher activation of the biofilm and cellulose iModulon, which can be helpful for protection from the environment, was observed under S starvation (Fig. 5).

Our comprehensive analyses can be utilized as a reference to understand many bacterial physiologies and their applications. For example, the common stationary phase features observed with *E. coli* may be conserved in other bacteria, including pathogens. Clinical characteristics, such as biofilm formation, can be studied by analyzing the expression level changes of homologous genes to relevant *E. coli* iModulon member genes. Furthermore, different patterns depending on limiting nutrients can be applied for bioproduction, given microorganisms are often challenged by nutrient starvation to redirect metabolism toward production of a desired chemical. For example, N limitation has been applied to enhance microbial production of hydrogen (51), organic acids (52, 53), biofuels (54), biopolymers (55), etc.

Given sequencing of bacterial messenger RNA (RNAseq) developed rapidly over the past decade, and with protocol improvement and automation (56), we can now generate multiple time series of transcriptomes to help us reveal how their composition changes during the transition from one physiological state to the next. Analyzing a time series of transcriptomes has been informative, with sporulation in *Bacillus subtilis* (6) and adaptation of *Staphylococcus aureus* to human plasma (57), providing illustrative examples. In this manner, ICA will be effectively and widely applied to understand industrially or clinically important microbial behaviors. These efforts will expand our knowledge to biological regulatory networks, guiding synthetic biology to redesign natural biological systems.

## MATERIALS AND METHODS

### Culture conditions

All cultures of *Escherichia coli* K-12 MG1655 were performed at 37 ℃ in a minimal M9 medium. The M9 minimal medium contains 47.8 mM Na_2_HPO_4_, 22 mM KH_2_PO_4_, 8.6 mM NaCl, 18.6 mM NH_4_Cl, 2 mM MgSO_4_, 0.1 mM CaCl_2_, and 2 mL/L Sauer trace element solution. For a minimal M9 medium, glucose was added as a carbon source at a concentration of 2 g/L. The Sauer trace element solution (100×) contains 1 g EDTA, 29 mg ZnSO_4_.7H_2_O, 198 mg MnCl_2_.4H_2_O, 254 mg CoCl_2_.6H_2_O, 13.4 mg CuCl_2_ and 147 mg CaCl_2_. For the N-limited condition, the NH_4_Cl concentration was reduced to 1.2 mM (a 16-fold decrease). For the S-limited condition, the MgSO_4_ concentration was reduced to 0.03 mM (a 67-fold decrease), with 1.97 mM MgCl₂ added to compensate for the reduced Mg^2+^.

*E. coli* K-12 MG1655 on an LB agar plate was inoculated into 10 mL of the M9 medium and grown overnight at 37 ℃. The following day, the cultures were re-inoculated into 100 mL of the same medium at 0.1 of an optical density of 600 nm (OD_600_). When OD_600_ reached approximately 1.0, cells were centrifuged and washed three times with a 75 mM potassium phosphate buffer. Washed cells were inoculated to 500 mL of the minimal M9 medium in 2 L flasks, and OD_600_ was regularly monitored. Similarly, washed cells were inoculated into N-limited and S-limited conditions. For gene expression profiling, 3 mL of cell cultures were taken at six time points (3, 4, 6, 8, 12, and 24 h), representing different phases of growth after inoculation and immediately mixed with 6 mL of RNAprotect Reagents from Qiagen (Hilden, Germany). Two independent samples were collected for each time point under each condition. All samples were stored at −80℃ until library preparation.

### Gene expression profiling *via* RNA-Seq

Total RNA was extracted by using a Quick-RNA Fungal/Bacterial Kit from Zymo Research (Irvine, CA). Ribosomal RNA was depleted by following the RiboRid protocol that utilized hybridase thermostable RNase H and anti-ribosomal RNA oligonucleotides (58). RNA-sequencing libraries were prepared using a Kapa Hyperprep RNA kit from Illumina (San Diego, CA). Pair-end sequencing was performed using an Illumina NovaSeq 6000 at the UC San Diego IGM Genomics Center with 50 × 2 cycles. Raw read files were processed with the reference genome of *E. coli* K-12 MG1655 (NC_000913.3) by running an in-lab pipeline (https://github.com/avsastry/modulome-workflow) developed for analyzing microbial gene expression profiles (59) to obtain transcripts per million (TPM) values for each gene. Differentially expressed gene analysis was conducted by using Deseq (60). The differential gene expression was defined as the fold change >2 with p_adj_ <0.05.

### Independent component analysis of the gene expression profiles

The ICA analysis and data visualization were conducted using the Pymodulon package (3). A new iModulon structure was calculated as previously reported (3, 61). Briefly, the [**X**] matrix of the gene expression profile was generated by concatenating PRECISE-1K with the normalized TPM values of the 36 samples by the PRECISE-1K reference condition TPM values. Robust independent components (ICs) were obtained by performing FastICA (62) using Scikit-learn. This process decomposes the [**X**] matrix into [**M**] and [**A**] matrices, where [**M**] contains gene weights and [**A**] contains the activity for each IC. The optimal dimension was 375 by utilizing the OptICA method (61), resulting in 247 iModulons. The newly calculated iModulon structure was compared with the existing structure, and the same names were assigned for similar iModulons, which displayed Pearson correlation coefficients greater than 0.7. Less similar iModulons were named based on known regulator names if enriched with a false discovery rate (FDR) of 10^−4^. Several iModulons, including the remaining unnamed iModulons, were characterized or named manually (*e.g.*, ppGpp-1, YciT) based on regulator names, operon names (*e.g.*, *yddABC*, *ybiIXL*), etc. The activities of PRECISE-1K iModulons in the 36 new RNA-seq samples were inferred by using the infer_activities function based on gene weights in the M matrix of PRECISE-1K (9). Explained variance for a given iModulon was calculated by using the explained_variance function.

## Data Availability

Raw RNA-seq files were deposited to NCBI Gene Expression Omnibus (https://www.ncbi.nlm.nih.gov/geo/) under the accession number GSE226643. Jupyter Notebook files for analyzing the data and generating figures are available from GitHub (https://github.com/hyungyulim/Ecoli_transition_stationary).

## SUPPLEMENTAL MATERIAL

Supplemental figures msystems.01315-24-s0001.docx Figures S1 to S9.

ASM does not own the copyrights to Supplemental Material that may be linked to, or accessed through, an article. The authors have granted ASM a non-exclusive, world-wide license to publish the Supplemental Material files. Please contact the corresponding author directly for reuse.
