# Supplementary material for: Revealing systematic changes in the transcriptome during the transition from exponential growth to stationary phase
Source: mSystems. 2024 Dec 23;10(1):e01315-24. doi: 10.1128/msystems.01315-24 (PMC11748552; doi:10.1128/msystems.01315-24)
Supplement: Supplemental figures — Figures S1 to S9. [file msystems.01315-24-s0001.docx]

Supplementary Information of
Revealing systematic changes in the transcriptome during the transition from exponential growth to stationary phase

Hyun Gyu Lim^1,2,3,*^, Ye Gao^2,4^, Kevin Rychel^2^, Cameron Lamoureux^2^, Amy Lou^2^,
Bernhard O. Palsson^2,3,5,*^

^1^Department of Biological Sciences and Bioengineering, Inha University, 100 Inha-ro, Michuhol-gu, Incheon 22212, Korea

^2^Department of Bioengineering, University of California, San Diego, 9500 Gilman Dr. #0412, La Jolla, CA 92093-0412, USA

^3^Joint BioEnergy Institute, 5885 Hollis Street, fourth floor, Emeryville, California 94608, United States

^4^The Second Hospital of Shandong University, Beiyuan Street 247, Jinan, Shandong 250033, China

^5^The Novo Nordisk Foundation Center for Biosustainability, Technical University of Denmark, Building 220, Kemitorvet, 2800 Kgs. Lyngby, Denmark

*To whom correspondence should be addressed.

**Contacts**

(Bernhard O. Palsson) palsson@ucsd.edu

(Hyun Gyu Lim) hyungyu.lim@inha.ac.kr

# Supplementary Figures

##
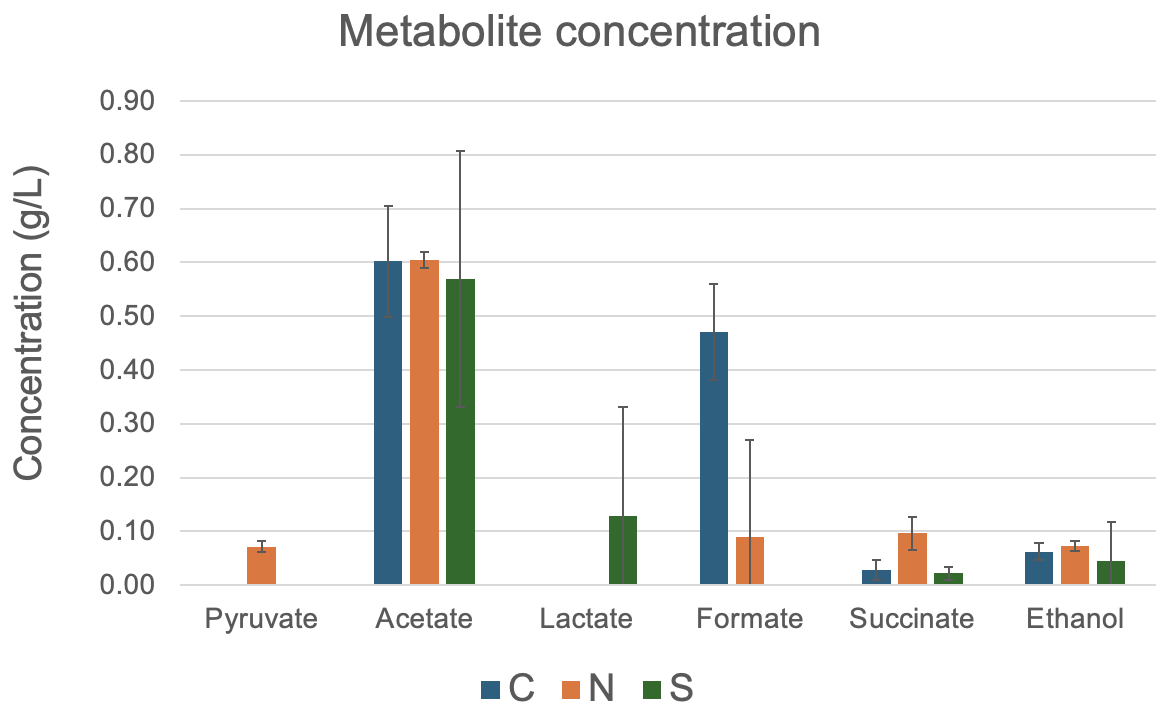


## Figure S1. Metabolic accumulation in the C, N, and S-limiting conditions

Maximum concentrations of byproducts accumulated during the culture. *y*-axis indicates metabolite concentrations (g/L). The error bars indicate the minimum and maximum values of the two replicates.

##

##
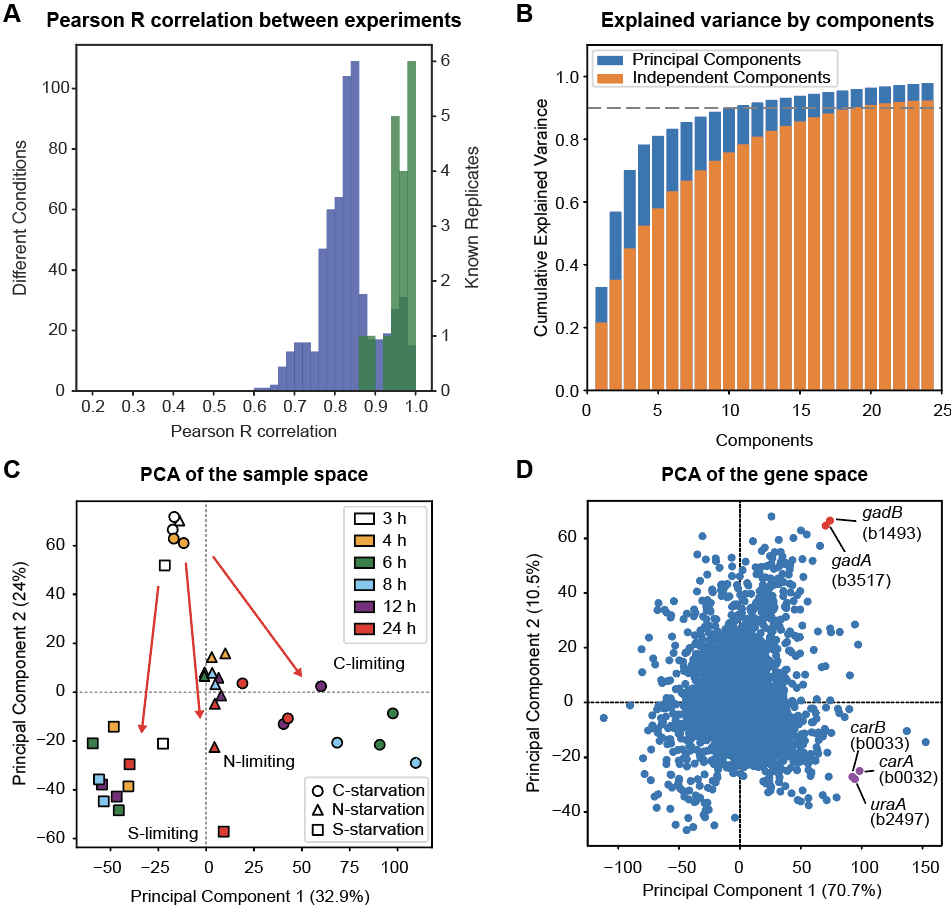


## Figure S2. PCA of the new transcriptomic data.

(**A**) Pearson correlation between known replicate samples (green) and between random pairs (blue). (**B**) Cumulative explained variance by the number of principal components or independent components. (**C** and **D**) PCA analysis of (**C**) the 36 samples and (**D**) the 4,257 genes in *E. coli* K-12 MG1655.

##

##


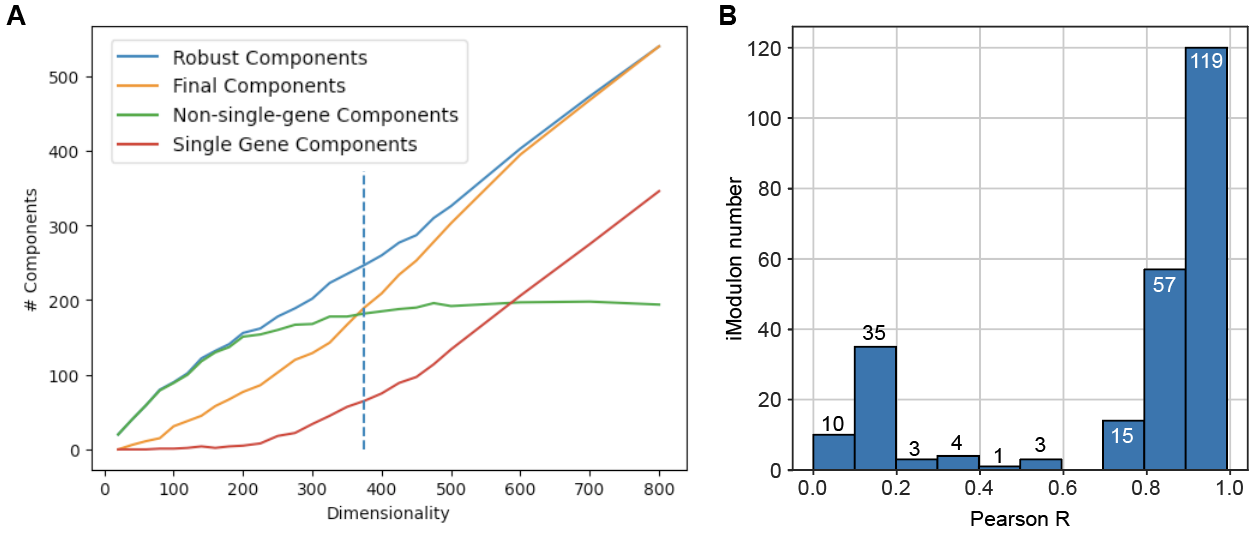


## Figure S3. Independent component analysis of the consolidated dataset of PRECISE-1K plus the 36 transition dataset

(**A**) The choice of the optimal dimensionality to determine the number of independent components. The optimal dimension was chosen as 375, where the number of non-single gene components reached a plateau. Determination of the optimal dimension (**B**) A histogram of Pearson correlation coefficients of gene weights for conserved PRECISE-1K iModulons.


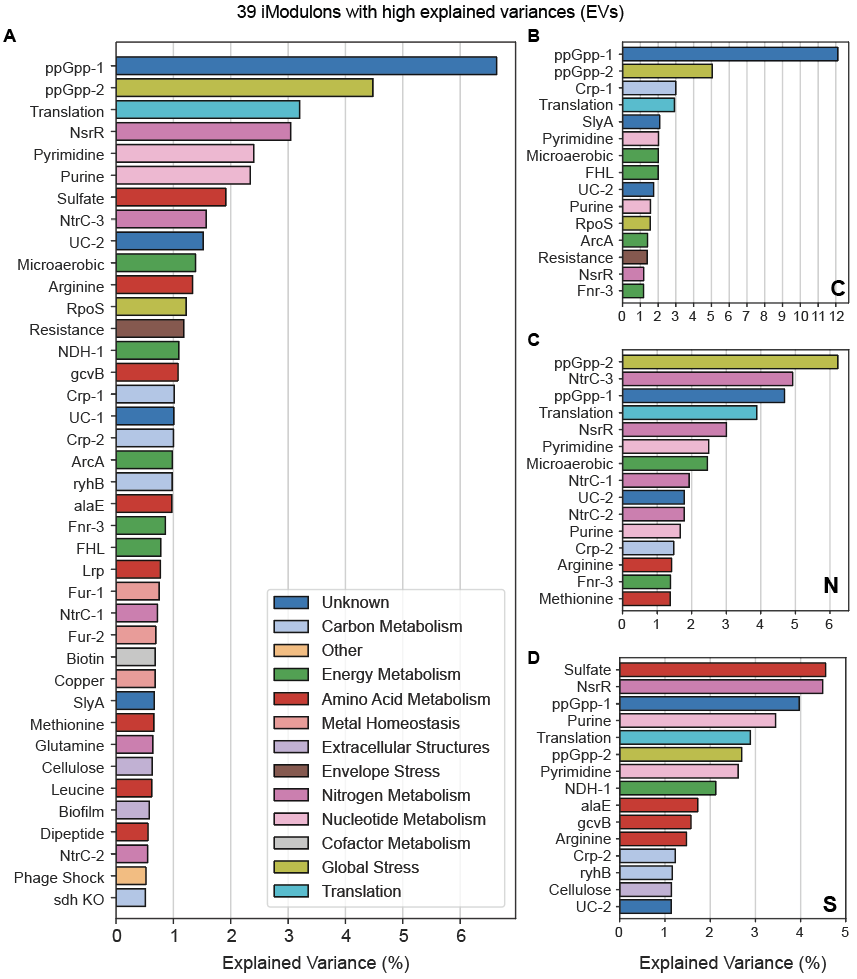


## Figure S4. iModulons with the highest explained variance for each starvation condition.

(**A**) Explained variances of the 39 iModulons with greater than 0.5% of the explained variances of the stationary phase dataset. (**B-D**) The top 15 iModulons with the highest explained variance in (**B**) C (**C**) N, (**D**) S-limiting conditions, respectively. The color legend indicates iModulon functions.


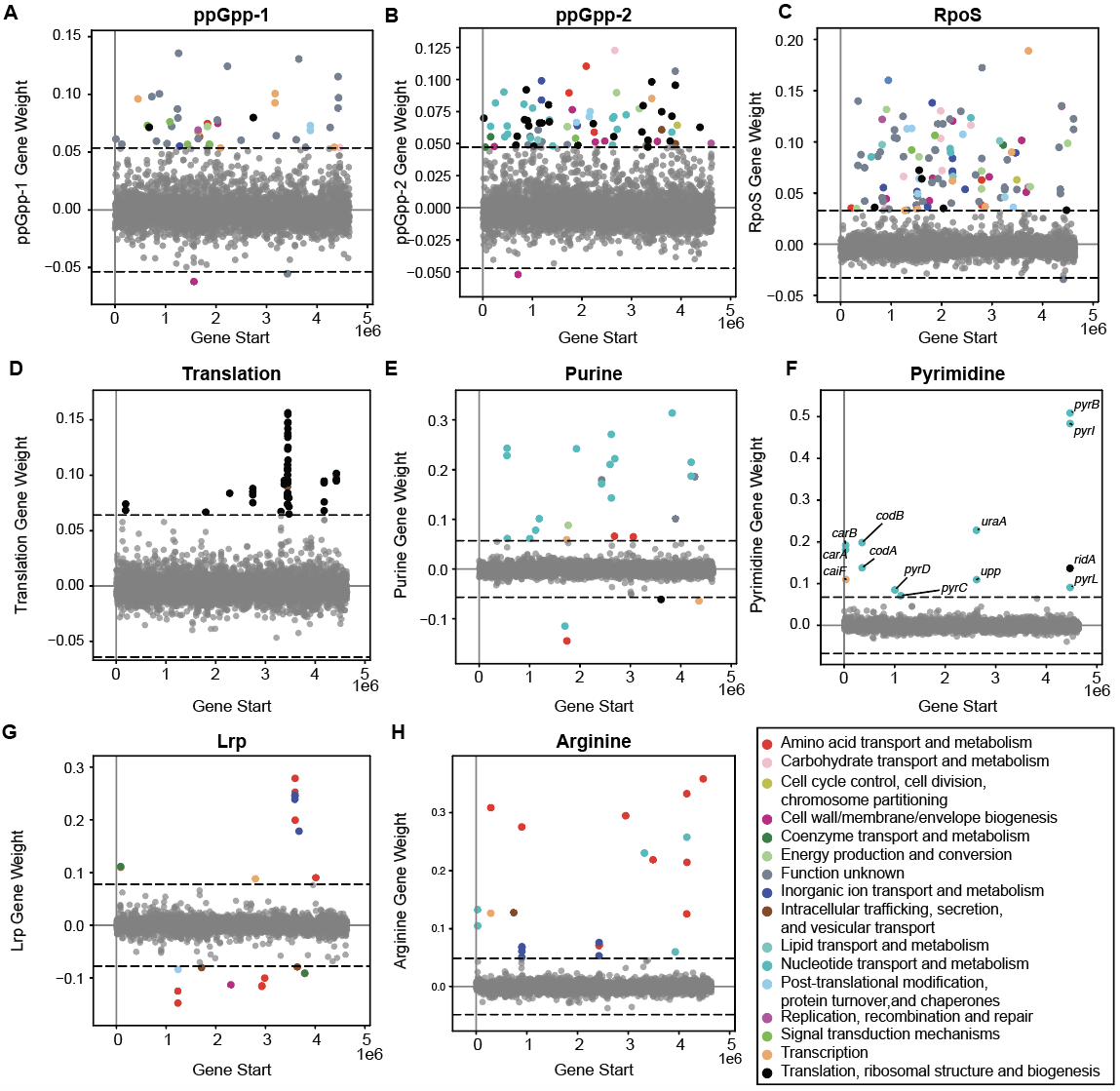


## Figure S5. Gene weights of iModulons related to stationary-phase responses

##

##

##
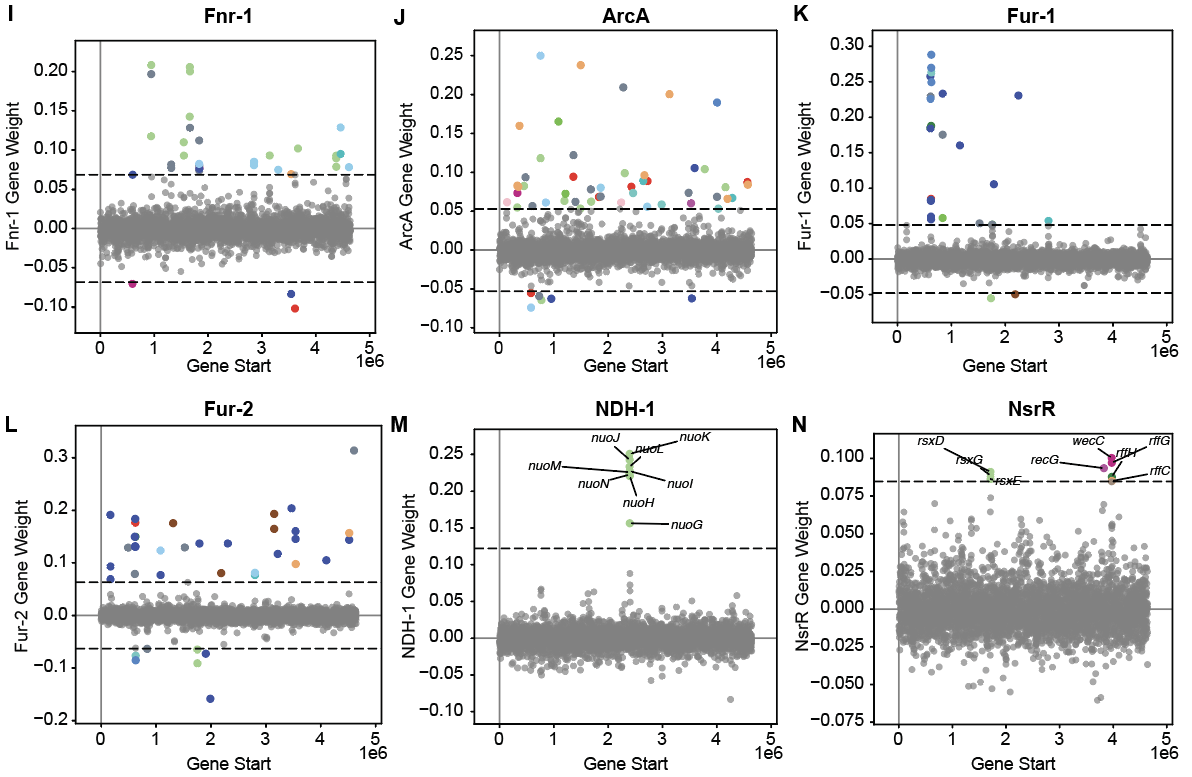


## Figure S5. Gene weights of iModulons related to stationary-phase responses (continued)


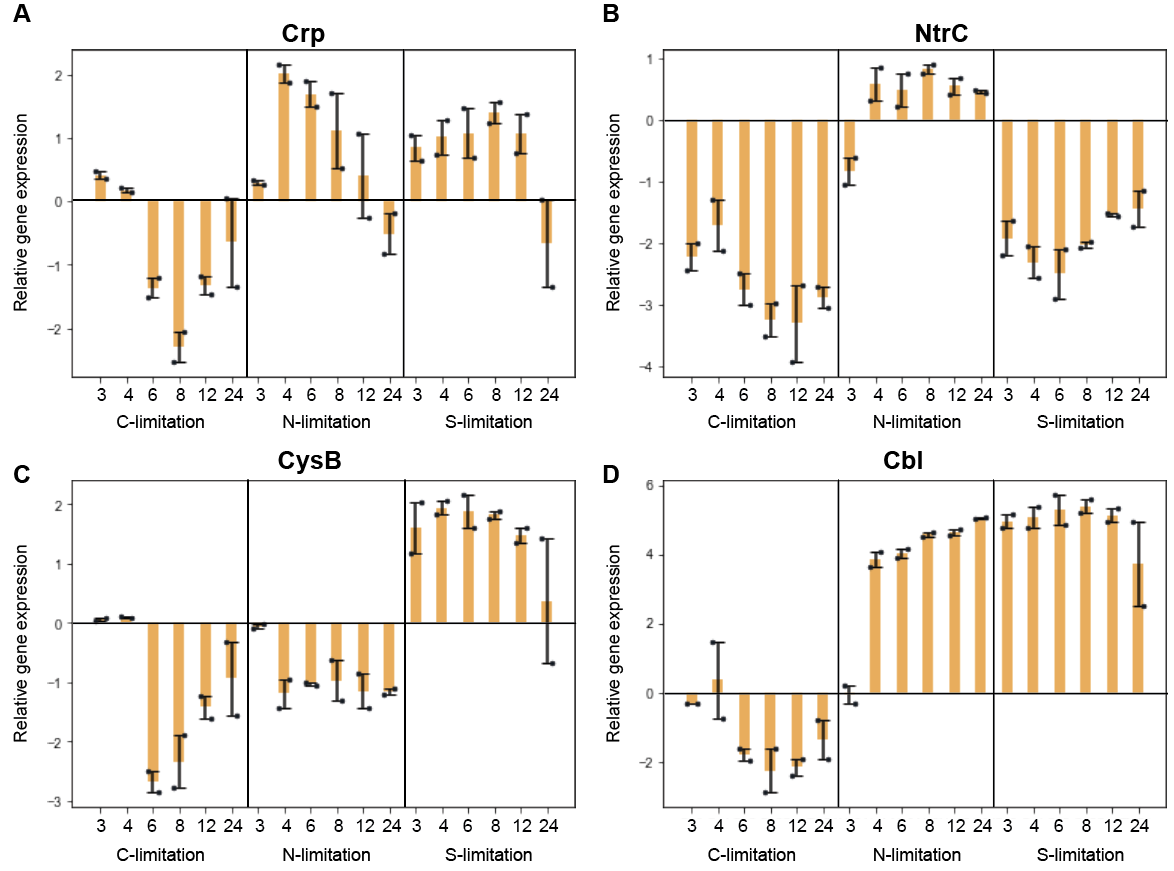


## Figure S6. Expression of global regulator genes

(**A-D**) Relative expression of encoding regulators, (**A**) Crp, (**B**) NtrC, (**C**) CysB, and (**D**) Cbl. The expression was normalized by the expression levels of the reference condition of PRECISE-1K.


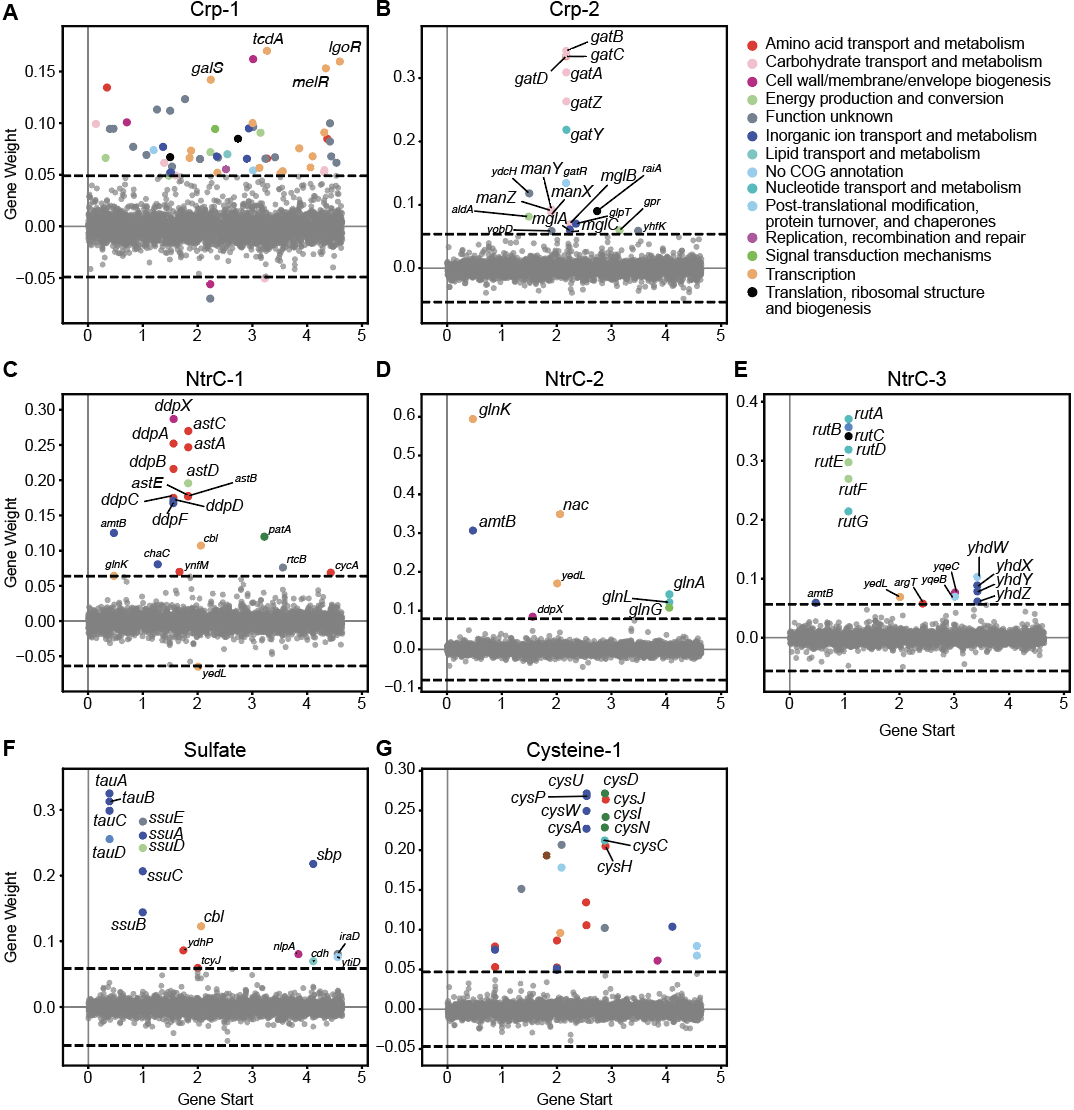


## Figure S7. Gene weights of C, N, S-metabolism related iModulons

*x*-axis and *y*-axis indicate gene locations and gene weights, respectively.


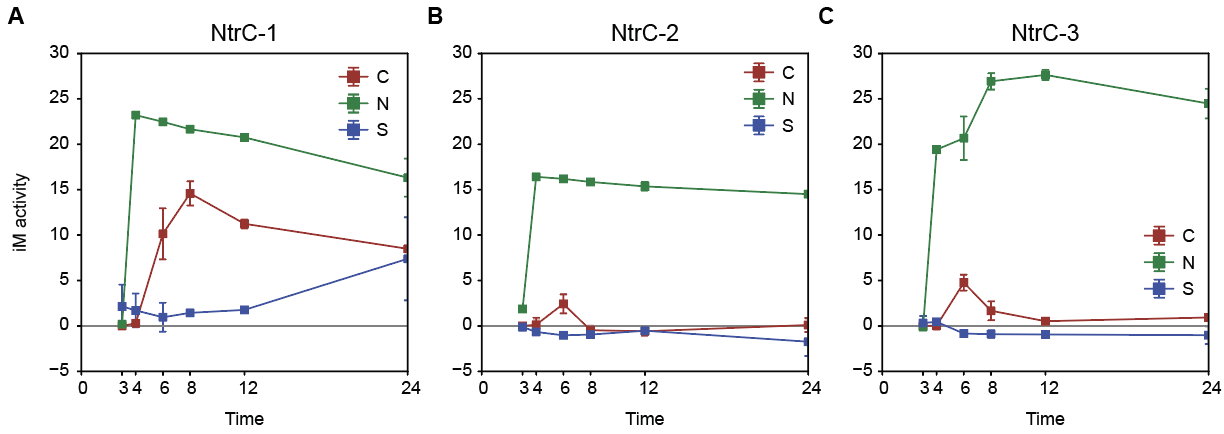


## Figure S8. Time-course activities of the three NtrC-related iModulons (NtrC-1, NtrC-2, NtrC-3)

*x*-axis and *y*-axis indicate time (h) and iModulon activity, respectively. The error bar indicates the two values of biological duplicates.

##
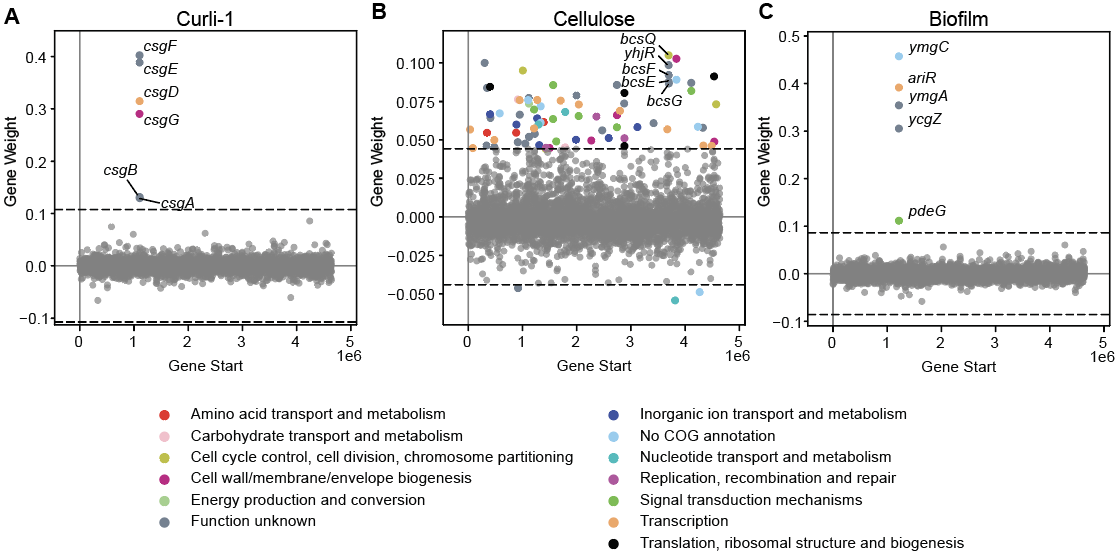


## Figure S9. Gene weights of the Cellulose, Curli-1, and Biofilm iModulons

*x*-axis and *y*-axis indicate gene locations and gene weights, respectively.
